# Brain stimulation and elicited memories

**DOI:** 10.1007/s00701-022-05307-6

**Published:** 2022-07-08

**Authors:** Rickard L. Sjöberg

**Affiliations:** 1 Department of Clinical Science, Umeå University, Umeå, Sweden; 2 Department of Clinical Science, Neurosciences, Umeå University, S901 85 Umeå, Sweden

**Keywords:** Electric brain stimulation, Awake craniotomy, Deep brain stimulation, Memory, Experiential phenomena

## Abstract

**Background:**

Since the late 1930s, electric brain stimulation (EBS) in awake patients has been known to occasionally elicit patient descriptions of a form of memory flashbacks, known as experiential phenomena. One understanding of these sensations are as caused by an augmentation of the capacity for memory retrieval. However, an alternative hypothesis holds that memory flashbacks during EBS are “synthetic constructions” in the form of mental events, falsely interpreted as memories.

**Methods:**

A critical narrative review is used to discuss the false memory hypothesis in relation to the current empirical literature and source attribution theory.

**Results:**

EBS as well as situational demands in the form of interaction between patient and neurosurgeon may both lead to the creation of mental events and influence their interpretation in a way that may create false memories. The false memory hypothesis provides a potential explanation for several apparent inconsistencies in the current literature such as (a) the fragmented nature of experiential reports, (b) the ability of EBS to induce memory retrieval errors in controlled studies, (c) that Penfield’s elicitations of experiential phenomena are so rarely replicated in the modern era, and (d) the limited utility of techniques that elicit experiential phenomena in the treatment of memory disorders.

**Conclusions:**

The hypothesis that experiential phenomena may largely be “synthetic constructions” deserves serious consideration by neurosurgeons.

In 1934, Wilder Penfield [42] applied what he would later describe as a “gentle electrical stimulus” to the right superior temporal gyrus of a 14-year-old girl known by the initials J. V. This occurred as part of an awake craniotomy at the newly established Montreal Neurological Institute (MNI) and the purpose was to elicit a specific dream-like aura of anticipatory fright (related to a childhood memory) that always preceded her disabling and therapy-refractory epileptic seizures.

During the surgery, it was possible to successfully reproduce the aura by means of stimulation of the parietal cortex. But as the cortical mapping procedure was further carried on along what was identified as the extension of the superior temporal gyrus, the patient also reported living through other experiences that were not part of her typical semiology.

“These stimulations caused the patient to cry out that she heard a large number of people shouting. Once she said: “They are yelling at me for doing something wrong; everybody is yelling.“ On inquiry she said she could hear her mother and brothers.” (42 p. 432).

This was presumably the first example of what Penfield was later to describe as an “experiential phenomenon” elicited by direct electric brain stimulation (EBS) in a neurosurgical setting. He would later define this as a phenomenon that would occur after EBS would “cause the conscious patient to be aware of some previous experience.” (43 p1719; 40). A phenomenon that could in present-day terminology be described as an induced retrieval or recovery of an episodic memory.

Since then, according to a systematic review and online database published by Curot et al. [6], similar phenomena, apparently related to some form of memory retrieval, have been observed in approximately 112 patients in the published literature. One recent example is a study presented by Deeb et al. [9] who stimulated the fornix of 42 patients with Alzheimer’s disease, participating in a clinical trial. Here the authors report having elicited experiential phenomena in 20 of these patients. These authors present an apparent dose-dependent increase of clarity of the reminiscences describing that:

“… a patient’s recollection at 7-V stimulation evolved from a generic notion of “helping a guy find something on his property” to, at 10-V stimulation, remembering “that this event occurred at night around Halloween” (9 p. 783)

Most instances of EBS-induced memory recovery have been elicited through stimulation of either the superior temporal gyrus or the hippocampus and/or parahippocampal structures, including the amygdala, entorhinal cortex, parahippocampal and fusiform gyri, and fornix [7]. This means that this phenomenon appears to add a tantalizing dimension to our theoretical understanding of the role these medial temporal structures and their interconnected networks play in declarative and episodic memory [2, 35, 47].

However, on the one hand, the role of these structures appears to be about normal memory function [58]. But on the other hand, much of the literature on which our understanding of the neural substrates of memory is based is also a literature on memory malfunction. Perhaps, the most iconic example of this is the demonstration of a complete loss of declarative memory in patient HM after the bilateral neurosurgical removal of his medial temporal structures [37, 48]. Another set of similarly intriguing findings is provided by studies of confabulation of episodic memories after injury to brain regions that are connected to the hippocampus [8].

The academic discussion of EBS-induced memory recoveries such as those described by Penfield almost 90 years ago parallels this dichotomy. Whereas most neurosurgeons that have observed these phenomena have tended to interpret them as some form of augmentations of normal memory function, other influential critics have chosen to interpret them as forms of memory malfunction. Whereas a definitive resolution of this debate is not possible without more carefully controlled studies, the purpose of the present review is to provide an update on the latter of these hypotheses considering developments during the most recent decades.

## Explanations for experiential flashback-like experiences during EBS

As described above, Penfield used the term experiential phenomena, to describe EBS-induced memory recoveries in the form of the patient becoming “aware of some previous experience” and contrasted this with “interpretive responses” in which brain stimulation influenced the way stimuli were subjectively perceived. However, EBS has also been known to induce a range of other complex mental phenomena, involving volition, perceptual, mnemonic, and affective features [11, 17, 43, 51]. In discussing such effects, some neurosurgeons have used broader definitions of these phenomena that encompass all of these aspects [11, 17]. For the sake of simplicity, the term experiential phenomena will however be used here in a way consistent with the original definition.

Penfield’s own interpretation of these phenomena was that the process elicited by the stimulation was “as though a wire recorder, or a strip of cinematographic film with sound-track, had been set in motion within the brain,” in the form of an experiential sequence, picked out of the patients own past [43, 44]. This interpretation has been further developed by later generations of neurosurgeons to suggest that these phenomena are the results of EBS-induced activation and enhancement of the neural systems and networks that form the substrate of the memory retrieval process [3, 19, 28]. Memory retrieval is, according to this theory, switched into high gear by the stimulation in a way that goes beyond what the stimulated brain can control [16, 29].

This explanatory model together with Penfield’s claim that what he found was “a permanent record of the stream of consciousness in the human brain” has important implications for our understanding of the way memory works and our understanding of the permanence of stored information in the human brain. That is, contrary to the views dominating the contemporary scientific understanding of memory, Penfield’s model appears to suggest that memory is stored in a permanent and fixed way rather than being reconstructed at the time of retrieval [1, 32]. In addition, from a neurosurgical clinical point of view, the most important consequence of the theory is that it provides an important part of the theoretical rational for clinical trials of chronic EBS with implanted electrodes as a treatment for disorders of memory [14, 19, 21, 29].

However, during the 1960s, as Penfield’s results were being widely disseminated, for instance in the context of introductory psychology courses, an influential alternative explanatory model for experiential phenomena, based on mainstream cognitive science, was introduced. According to this model, these phenomena should primarily be understood as mental events that are erroneously interpreted as memories [33, 40].

In a classical textbook (that has often been credited as a foundational document of the “cognitive revolution” in scientific psychology), Ulric Neisser lays out this argument and a critique of Penfield’s explanations in the following way:

“…there are three steps in Penfield’s argument although he does not make them explicit: (a) These images must be reproductive memories (rather than fantasies) because the patient experiences them as familiar; (b) they must be accurate (rather than confabulated) because they are so vivid and subjectively real; (c) the “record of the stream of consciousness” must be complete (rather than fragmentary) because it evidently includes trivial events.

It seems to me that all three inferences are unjustified; (a) the feeling of familiarity may be unjustified as it often is in daily life; (b) in some subjects, hypnagogic imagery is equally vivid but obviously does not represent actual recall; (c) the fact that some events are remembered hardly proves that no events are forgotten. […] the content of these experiences is not surprising in any way. It seems entirely comparable to the content of dreams, which are generally admitted to be synthetic constructions and not literal recalls.” (40, p. 149)

While acknowledging that Penfield’s electrodes may have had an effect in eliciting the experiential reports, Neisser argues that they may have touched on the “mechanisms of perceptual synthesis.” That is, to put it more bluntly, the EBS may have distorted the memory process by an artificial contribution to the creation of false memories.

Assuming that there is a chance that Neisser’s hypothesis could be right, how exactly could a false memory have been created in this kind of neurosurgical context and how would this possibility affect our understanding of some of the key points of confusion in the current literature?

## False memories as errors of source attribution

Imagine a person who, as part of a screening for cognitive disorders, such as Alzheimer’s disease, performs a test such as the Mini-Mental State Examination [13]. At one stage, the examiner reads three words to the patient, Rabbit, Watch, Truck, and asks the patient to repeat them which he does accurately. The patient is then told to memorize the words because one of the tasks will be to repeat them again, later. As the time comes for the patient to do this, he is able to repeat the first two words (Rabbit, Watch) accurately but hesitates when coming to the last one (Truck). Memory seems hazy. Eventually, the word bicycle pops up in his mind. Is this mental representation, a memory of the examiner saying the word? After some hesitation and after examining the characteristics of the mental representation, the patient decides that this is the case. Once the decision is made, the patient finally answers:

- I also remember you saying the word “bicycle”.

The answer is obviously wrong, caused by the fact that the patient erroneously interpreted a mental representation of the word bicycle as a memory of the examiner saying the word during a specific task. This kind of false memory is known in the research literature as a source attribution error [23, 24].

The central claim made by source attribution theory is well illustrated by contrasting it with a slightly caricatured version of Penfield’s theory on human memory, according to which memory is like a library of video films with tags on them describing when, where, and how the tapes were recorded. In contrast to this, source attribution theory claims that specific memory episodes do not come with tags. Instead, an attribution of the source of the memory is made during a memory reconstruction that occurs at the time of retrieval. This attribution is largely made based on characteristics of the mental representation at hand. So for instance a mental representation in the form of a word would, if it also included memory traces of another person’s voice, be much more likely to be attributed to a memory of that person saying the word than if the trace of that person’s voice was not part of the representation. Such source attributions are often made unconsciously and automatically but may, especially when the process is difficult also be made consciously based for instance on metacognitive reasoning. Such conscious metacognitive reasoning might, for instance, help you decide that a mental image of yourself meeting your grandpa will probably not represent a real memory if you know that your grandpa died before you were born [22, 25].

Source attribution theory has during the latest decades become an important tool for understanding how false memories may be created not only during simple cognitive testing but also in the context of clinical decision-making, forensic interviewing, or psychotherapy [5, 20, 30, 31, 53]. The theory has even been shown to be consistent with what is known about how false memories of having been kidnapped by Satanists were created in child witnesses during the period of early modern European witch persecutions [46, 49].

## False memories in the context of EBS

**Fig. 1:**
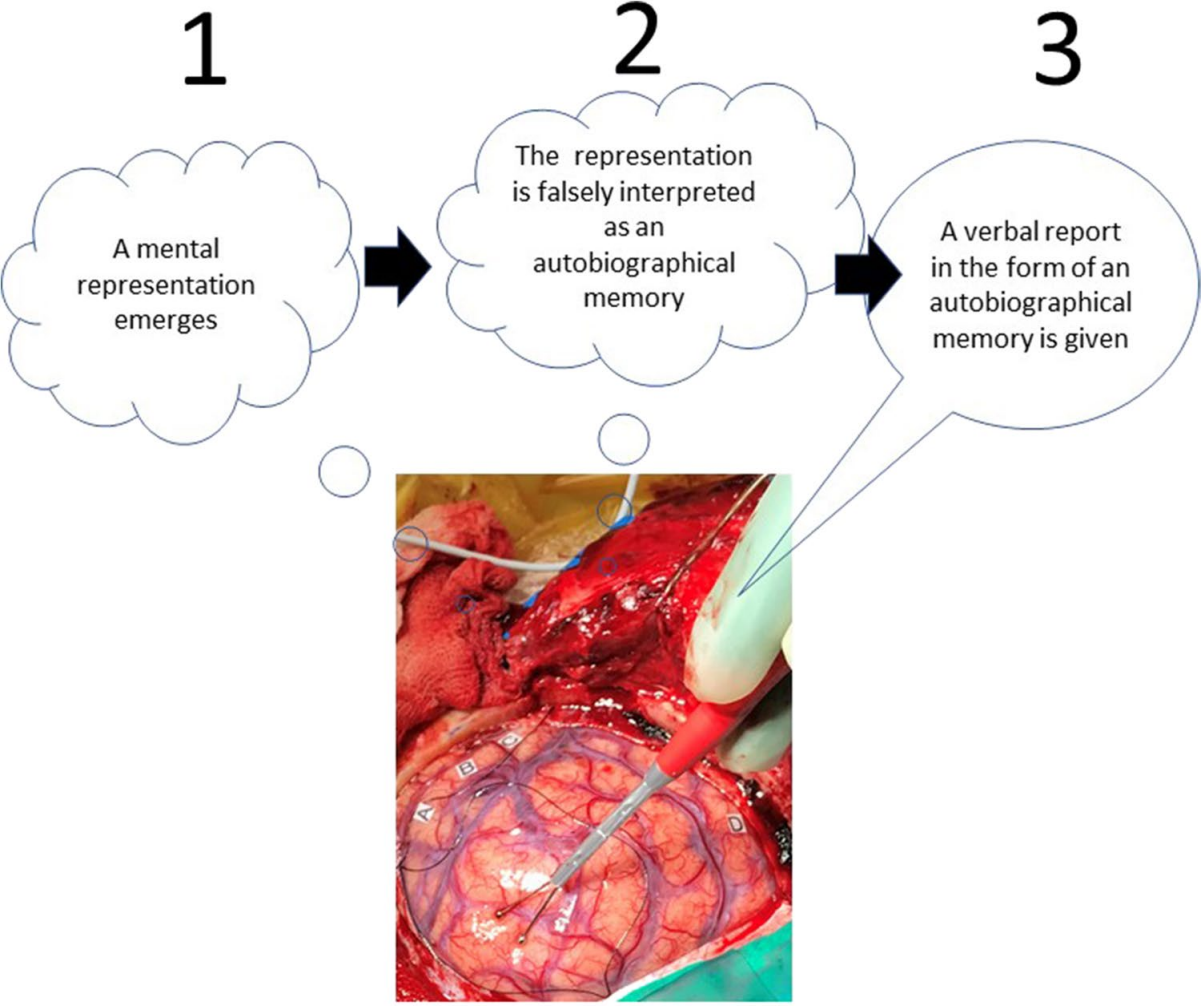
Postulated process by which introspective self-reports of experiential phenomena may be produced during electric brain stimulation (EBS) according to the source attribution hypothesis

**Fig. 2:**
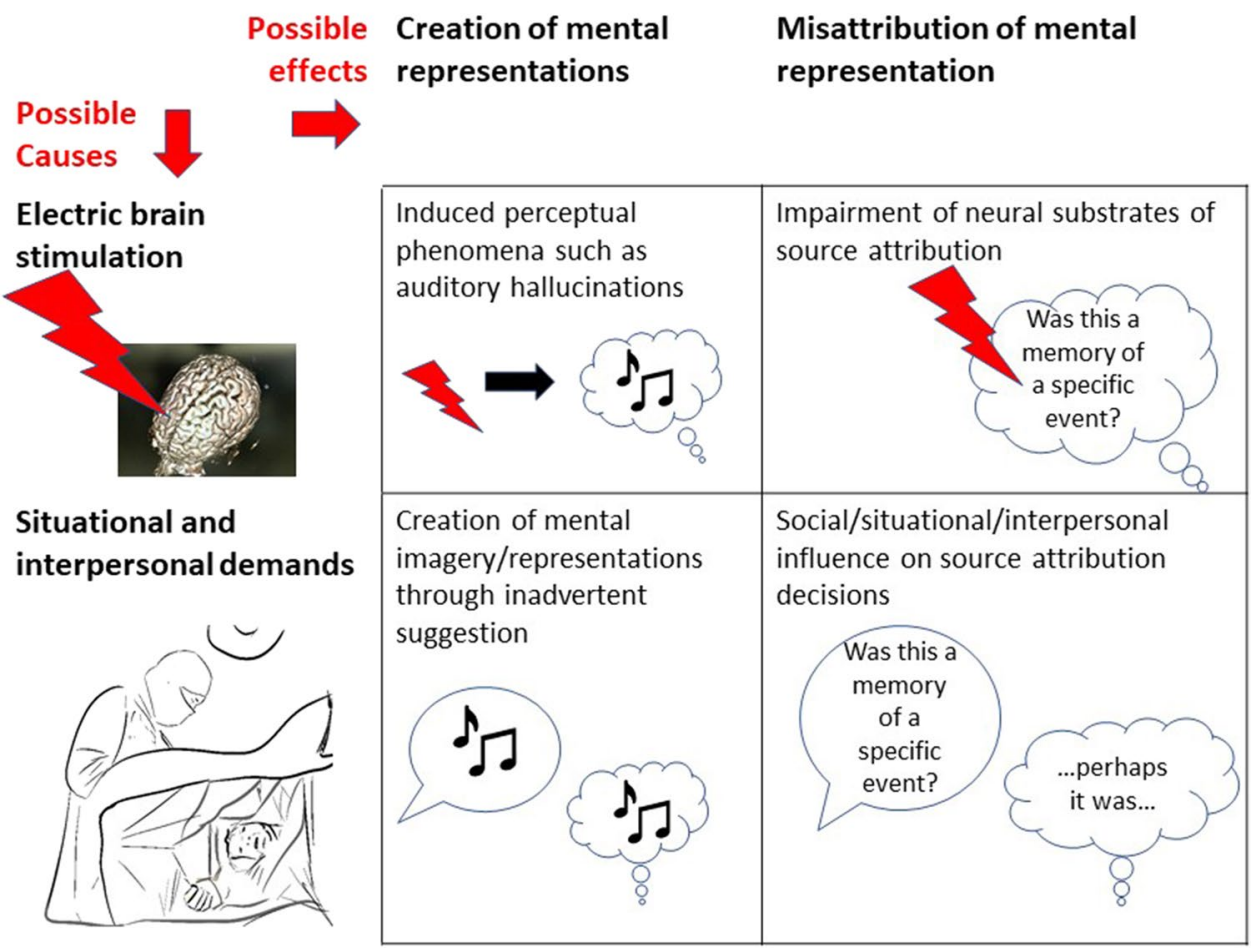
Examples of different types of possible source attribution errors in neurosurgical settings

In sum, a false report of a memory occurring in a neurosurgical setting can be described as a three-step process involving the emergence of a mental representation, the misattribution of this representation as an autobiographical memory, and eventually the verbal report, communicating the content of the attribution process (Fig. 1). When EBS is administered to a patient in a neurosurgical setting, there are at least two modalities through which the source attribution process may be influenced (Fig. 2).

First, there is a possibility of interpersonal, contextual, and situational influence, i.e., by “suggestion” [4]. That is, the situation, expressed instructions and expectations [54], and the way questions are asked [32] may either contribute to the creation of mental representations and/or the misinterpretation of certain mental representations as memories [30, 31].

Second, the electric stimulation itself may exert similar effects either by creating mental representations and/or by impairing the precision and capacity of the attribution of the source of such representations.

Finally, there is a possibility that these two modalities may interact. For example, there might be a possibility that contextual, situational, and interpersonal factors may prompt the patient to interpret EBS-induced artificial perceptions of sound as memories of real experiences. Or a mental representation might be induced by suggestion and the stimulation may make it harder for the patient to accurately attribute the source of this representation.

There is unfortunately no scientifically validated method that, with a high amount of precision, can distinguish verbal accounts of memories that are “synthetic constructions” from those that are literally true in the absence of independent documentation of what the right answer is [41, 56, 57]. Since no detailed documentation (for instance in the form of actual real videotapes of remembered events) exists for the experiential reports described in the literature, it is thus impossible to conclusively judge whether the memory enhancement hypothesis, the source attribution error hypothesis, or any other theoretical explanation for experiential phenomena is the right one. However, it is possible to examine some of the inconsistencies in the literature in the light of the source attribution hypothesis, and in most of the remaining part of this review, I will try to do so.

## The fragmented nature of experiential reports

In their comprehensive review of the literature on EBS-induced experiential phenomena, Curot et al. [6] found that most of the published cases were described in relatively sketchy and fragmented ways. Furthermore, the sensory and perceptual modalities referenced in these descriptions appeared to vary systematically depending on the site that had been subject to stimulation. Perhaps, the most striking feature of this finding was that all reports that described what Curot et al. [6] classified as “auditory semantic memories” (i.e., reliving or remembering the experience of hearing a piece of music, trying to figure out what it was, hearing people talking, shouting, etc.) were reported by patients after stimulation of the superior temporal gyrus or the insula adjacent to the superior temporal gyrus which are both cortical structures that define the borders of the primary auditory cortex. Curot et al. [6] concluded that this fragmentary nature of the experiential reports did not support Penfield’s theory according to which patients were reliving a complete experience of an event as if “the sights and sounds and thoughts of a former day” passed through his or her mind again (43 p 1719).

However, if experiential reports are understood as errors in the attribution of the source of EBS-induced perceptual phenomena such as auditory illusions, the data make more sense. The case JD might serve as an illustration of the phenomenon. That is, Penfield’s stimulation of her superior temporal gyrus is highly likely to have induced activity in her immediately adjacent primary auditory cortex. It seems reasonable to assume that such sensations could consciously or unconsciously have prompted JD to try to interpret and make sense of the signals from this area which can under normal circumstances only be caused by hearing sounds [46]. She eventually decides that she hears people shouting and after “inquiry” she says that she feels that she can discern the voices of family members. The fact that this episode is later related and described as a memory implies that she and her neurosurgeon eventually decided that this was the case. But the details describing how that decision, which made the source attribution complete, was made, do not appear to have been recorded.

Interestingly, this explanation would be consistent with observations made by other authors who have described auditory hallucinations elicited by stimulation of the lateral temporal lobe [2, 12]. So for instance Adelman-Gur et al. [12] described complex auditory hallucinations which “for the most part included voices without specific verbal contents” as induced primarily after stimulation of the superior and middle temporal gyrus.

## EBS-induced source attributions in controlled studies

So far, all published reports of EBS-induced experiential phenomena are retrospective and anecdotal which means that they do not constitute objective tests of the memories of patients under controlled circumstances for information which is known by the neurosurgeon. However, there is also a growing literature in which patients have answered objective and controlled memory tests while being subject to EBS. Most of these have concerned themselves with studying the effects of EBS during the encoding of memories, sometimes combined with continued stimulation during storage and retrieval [29, 35]. Here, results have been mixed and a detailed discussion of this literature goes beyond the scope of this review. However, there is also a part of this literature that has focused on EBS during retrieval only. Here results are relatively clear. EBS of brain regions known to be associated with the elicitation of experiential phenomena does not enhance memory when given under experimental conditions. Instead, this literature shows an increased amount of commission errors on memory tests [18, 26, 27]. One early, elegant example of this phenomenon is given in a study by Fried et al. [15] who studied the effects of EBS in the right perisylvian region during awake craniotomies on the ability to recognize faces as well as lines presented at different angles. Briefly, the stimulus was first presented to the participants and then, after a distraction task, the participants were asked to choose the right stimulus from multiple choices. The design was carefully balanced with regard to timing and place of the stimulation so that it was possible to statistically test if, and in such case where and when, EBS affected memory performance. The finding that is most interesting in the present context was that EBS in the superior temporal gyrus during the retrieval phase of the testing procedure significantly increased the tendency of participants to report that they remembered lines and/or faces that had in fact not been presented to them as part of the task at hand. Similar findings have been made for stimulation in the hippocampal area. As discussed above, these kinds of commission errors on memory test are typically understood as caused by mistakes in source attribution. The validity of this and similar findings are furthermore supported by the fact that temporal and particularly medial temporal areas are known to be involved in the source attribution processes from fMRI studies [38].

As discussed above, another feature of non-auditory EBS-induced experiential phenomena is that most of them have been observed after stimulation of medial temporal structures. The fact that pathological input into hippocampal and parahippocampal memory circuits appears to have the capacity to induce confabulated episodic memories [8] would also appear consistent with a false memory interpretation of these phenomena.

Explaining the fact that experiential phenomena predominantly occur in regions where EBS is known to elicit source attribution errors is difficult from the point of view of a memory enhancement hypothesis. From the point of view of the source attribution error hypothesis on the other hand, the patterns of observations are self-explanatory. The fact that EBS-induced experiential phenomena tend to occur after stimulation of sites where EBS is known to induce source attribution errors implies that EBS-induced experiential phenomena in fact *are* source attribution errors.

## Why are Penfield’s findings of experiential phenomena during awake craniotomies so rarely replicated in the modern era?

One of the findings that was recently highlighted by Curot et al. [7] is that experiential phenomena seem to occur with varying frequency in different settings. Most striking is that even though awake craniotomy with direct cortical stimulation is today widely used in glioma surgery as a method for obtaining maximal safe resection in or near eloquent areas [10, 39, 55], no examples of EBS-induced experiences in such settings have been presented during the latest half century. Curot et al. [7] discuss nine possible explanations for this remarkable fact. One of these, the idea that the difference may be caused by differences in interviewing techniques and the interest expressed by the surgeon in these kinds of phenomena seems particularly relevant when the issue is viewed from a source attribution error perspective. What Curot et al. [7] suggest is that surgeons who find these phenomena interesting may be more committed to questioning patients about them. This may in turn encourage patients to verbalize such experiences. Furthermore, as has been discussed above, it is well known that certain ways of expressing expectations as well as certain ways of shaping an investigative or diagnostic interview may at times be enough to induce source attribution errors [4, 20, 52]. That is, mental representations that could potentially be construed as memories from a self-experienced event may easily be invoked by expressed expectations and information given to the patient. Furthermore, if a trusted authority such as an expert on brain function expresses expectations that certain mental representations (for instance ones that occur during EBS) should be actual memories, this is highly likely to tilt the source attribution process of an individual in the direction suggested by the expert [31, 36].

The fact that the highest incidence of elicitation of the experiential phenomenon reported in any patient sample so far was reported within the confines of a study specifically targeting EBS and memory [9] may have several explanations. However, one of these is that expectations of memory-related experiences may have been more pronounced in this clinical group than in other published materials and another one that the study was performed in memory-impaired patients. Both these explanations would be consistent with a source attribution error hypothesis.

## The limited utility of techniques that elicit experiential phenomena in the clinical treatment of memory disorders

One important source of inspiration for the first phase II clinical trial of DBS as a treatment for Alzheimer’s disease was the observation that stimulation of the fornix in one patient treated for morbid obesity was able to induce experiential phenomena. In addition, on follow-up neuropsychological testing, the same patient showed significant improvement on the California Verbal Learning Test which was one of several memory tests that had been administered to the patient preoperatively. He also showed improved results on one aspect of memory testing for word pairs (tested with two different techniques) with stimulation on vs when tested with stimulation off [19].

The results of a prospective randomized clinical phase II trial of DBS as a treatment for Alzheimer’s disease apparently confirmed the ability of this treatment to elicit experiential phenomena [9]. However, no stable improvement of the intervention at the group level on controlled cognitive tests was seen [34].

The failure of a technique that elicits experiential phenomena to enhance memory under controlled conditions is obviously problematic for any theory that seeks to explain such phenomena as a result of memory enhancement. This study furthermore undermines the argument that the wrong target or wrong stimulation technique was applied. If the right target and stimulation techniques would be those that are associated with the elicitation of experiential phenomena, then we would have expected this study to succeed. From the point of view according to which experiential phenomena are understood as source attribution errors an explanation is easier. After all, there is always a risk that an individual uncontrolled case report could represent an atypical scenario. The source attribution error hypothesis suggests that this might have been the case here and that the randomized clinical trial shows the result that would have been expected if the applied method has either no effect on memory function or a detrimental one.

## Discussion

It is in our current state of knowledge virtually impossible to prove or disprove any detailed theory on the genesis of the so-called experiential phenomena that were for the first time described by Penfield in 1936.

However, the possibility originally proposed by Neisser in 1967 that experiential phenomena may be nothing but synthetical constructions has so far not been conclusively proven wrong by later observations. On the contrary, the explanatory power of the false memory hypothesis equals or even surpasses the memory enhancement hypothesis when it comes to explaining: (a) that experiential phenomena appear fragmented; (b) that the phenomenon occurs at sites where EBS is known to induce source attribution errors; (c) that the phenomenon occurs frequently in some clinical settings but not others; and (d) that techniques that elicit experiential phenomena have so far failed as therapeutic interventions for the treatment of memory disorders.

Of course, this should not be taken to imply that Neisser’s theory has been definitely proven true; that true reminiscences can never occur in neurosurgical settings; or that attempts by neurosurgeons to understand the neural mechanisms of memory and to apply such understandings to the treatment of memory disorders should be abandoned.

However, the fact that the source attribution error hypothesis can still be understood as a potentially valid alternative explanation to EBS-induced experiential phenomena should have some potentially important implications for contemporary neurosurgical discourse on memory issues. Most important, the fact that the false memory/source attribution perspective is still a viable alternative to the memory enhancement hypothesis suggests that neurosurgeons should apply the latter idea with caution.
